# Computational Study of a Copper-Catalyzed Synthesis of Fluoroalcohols from Alkylboranes and Ketones

**DOI:** 10.1021/acs.joc.5c01174

**Published:** 2025-09-03

**Authors:** Francisco A. Gómez-Mudarra, Gabriel Aullón, Jesús Jover

**Affiliations:** † Secció de Química Inorgànica, Departament de Química Inorgànica i Orgànica, 16724 Universitat de Barcelona, Martí i Franquès 1-11, 08028 Barcelona, Spain; ‡ Institut de Química Teòrica i Computacional (IQTC-UB), Universitat de Barcelona, Martí i Franquès 1-11, 08028 Barcelona, Spain

## Abstract

Fluoroalcohols are a class of organic compounds containing one or more fluorine atoms together with an alcohol group in their molecular structure. These fluorinated species have a wide range of applications due to their unique properties and are used in medicine and electronics. Herein, we propose a new synthetic procedure, promoted by a copper­(I) catalyst, for preparing fluoroalcohols from alkylboranes and symmetric ketones. The reaction has been computationally explored to propose a plausible mechanism, which allows identifying the rate-limiting step and quantitatively evaluating the electronic effects of each substrate on the overall reactivity. These DFT calculations suggest that the combination of electron-poor ketones with electron-rich alkylboranes produces the most efficient catalytic systems for preparing fluoroalcohols. Microkinetic modeling of the studied systems allows the prediction of the activation barrier limit to achieve fully functional reactions, and multilinear regression techniques provide a methodology to estimate the overall reaction barriers in a simple manner, opening the way for proposing new catalytic systems.

## Graphical abstract

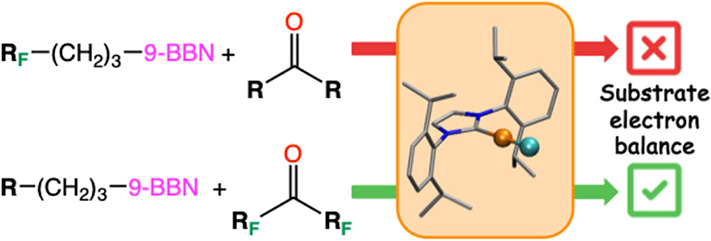

## Introduction

Fluorine, the smallest halogen and the most electronegative element, significantly alters the properties of the organic molecules when it is incorporated, giving particular physicochemical properties due to its electronic nature.^1,2^ Fluoroalcohols are a special class of organic compounds that contain an alcohol group along with additional fluorinated functional groups whose combined presence tends to produce unusual chemical behavior.^3,4^ In this line, fluoroalcohols have become an interesting class of solvents and their usage in organic synthesis has significantly increased.^5–19^ The most representative properties of fluorinated alcohols are their relatively high polarity, which arises from the difference in electronegativity between carbon and fluorine,^18^ and their solubility in a wide range of solvents,^20,21^ such as water,^22–26^ or other nonfluorinated alcohols.^27,28^ Fluoroalcohols tend to present rather low acidity,^29–31^ which turns the hydroxyl moiety into a stronger hydrogen-bond acceptor and weaker hydrogen-donor, resulting in a lower ability to form hydrogen bonds.^32–34^ This behavior agrees with the low vapor pressures and high volatility of fluorinated alcohols.^35–37^ In addition, the fluorination of alcohols increases their thermal stability, resulting in higher heat resistances than those found in their nonfluorinated analogues.^38–40^

In the context of scientific and technological research, fluorinated alcohols play a significant role, acting as essential compounds that propel advancements across multiple fields of study.^41–43^ In medicine, they act as inhalation anesthetics,^44,45^ contrast agents in magnetic resonance imaging,^46,47^ and drug carriers.^48–50^ In electronics, they can be employed for cleaning semiconductors,^51^ and also to enhance the efficiency of lithium batteries,^52^ fuel cells,^53,54^ and renewable energy storage systems.^55^ They are also used in the synthesis of polymers,^56–59^ surfactants,^60^ and pharmaceuticals,^41,61^ as well as in cosmetics,^62,63^ fire-fighting products,^64^ and high-performance lubricants.^65^ In addition, as an indication of their potential for technological innovation and advanced product development, a search in SciFinder-N for the term “fluorinated alcohol” (or “fluoroalcohol”), limited to the 21st century only, yields about 1300 articles and 1000 patents (i.e., about 52 and 40 per year, respectively). Owing to the great importance of fluorine-containing compounds, several synthetic routes have been explored in the last years.^2,66,67^ However, fluorination of organic molecules often requires special equipment and techniques, and the reagents are generally toxic and highly reactive, including the eventual release of hydrogen fluoride.

On the other hand, a significant progress has been made in developing metal-catalyzed systems for the incorporation of CO_2_ into organic molecules, both experimentally and theoretically.^68–71^ One such system, introduced by Skrydstrup and Nielsen, involves a dual hydroboration and copper-catalyzed carboxylation of substituted alkenes or alkynes using CO_2_ as the carbon source (Scheme 1).^72^ This method selectively forms new carbon–carbon bonds under mild conditions, proving highly effective in organic synthesis.

**Scheme 1:**
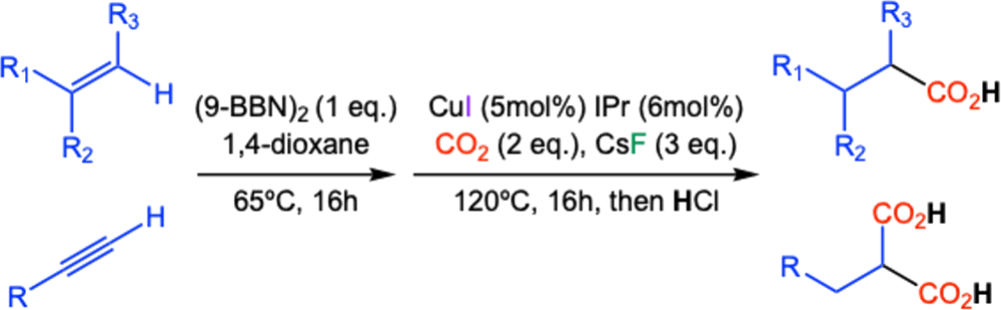
Copper-Catalyzed Hydroboration/Carboxylation of Alkenes and Alkynes with CO_2_ as Proposed by Nielsen and Skrydstrup^72^,^a^

a Adapted with permission from *ACS Catal.* **2017**, *7* (2), 1392–1396, Copyright 2017 American Chemical Society.

In this context, a computational study was previously carried out in our research group to corroborate the reported synthetic process (Scheme 2).^73^ In practice, this study involved the prospective exploration of the reaction between fluorinated alkenes and carbon dioxide to produce fluorocarboxylic acids as products, which may be of interest as synthons in medicinal chemistry or in the development of new materials. The DFT calculations indicate that the hydroboration of the starting alkene with 9-borabicyclo[3.3.1]­nonane (9-BBN) tends to mostly produce the anti-Markovnikov product, which can afterward be carboxylated with CO_2_ in the presence of the copper catalyst. The computed DFT reactions suggest that the proposed fluorinated carboxylic acids may be prepared following this synthetic route.

**Scheme 2:**
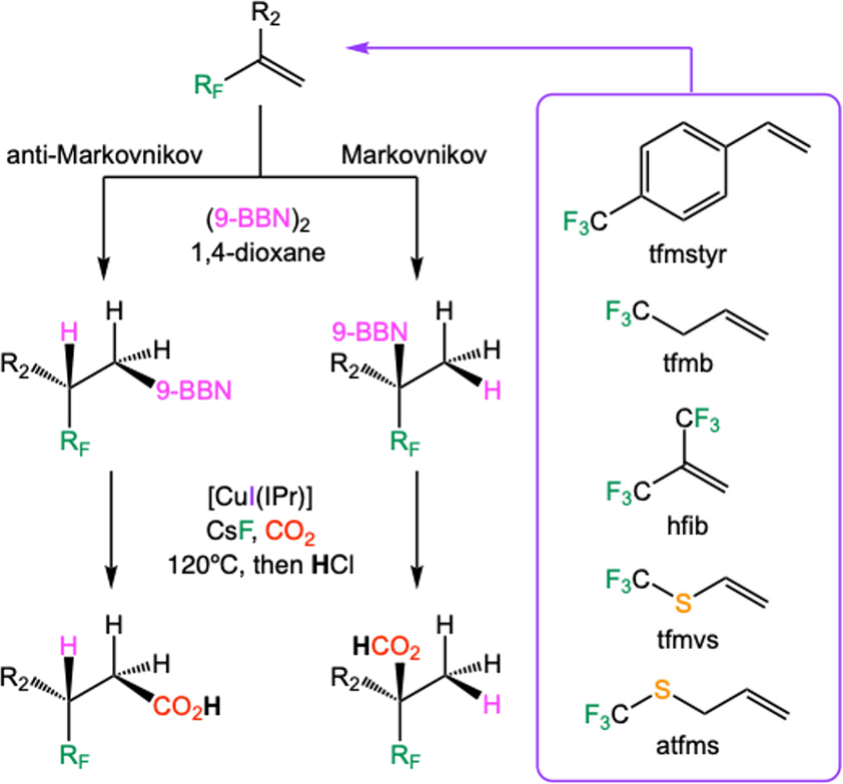
Computational Study of the Hydroboration/Carboxylation Sequence for Different Starting Fluorinated Alkenes with CO_2_ ^a^

a Adapted with permission from *Eur. J. Org. Chem.* **2022** (2), e202101243, Copyright 2022 Chemistry Europe.

In the present study, we have modified the original approach by replacing carbon dioxide with symmetric ketones with the aim of obtaining fluoroalcohols. Using ketones instead of carbon dioxide has some interesting advantages. For example, ketones are readily available materials with significant structural diversity that can expand the molecular structure of fluorinated alcohols. Additionally, ketones are good electrophiles in which the carbonyl group may resemble the basic structure of CO_2_, providing a similar reaction pattern for C–C bond formation. In this work, the hydroboration stage of the alkene with 9-BBN has not been calculated because in our previous report, which used the same starting species, it was found that the anti-Markovnikov intermediate was always the major product of this process. Therefore, only the copper-catalyzed C–C bond formation process has been computed using alkylboranes (R_F_-9-BBN) as starting materials, which should be preferentially formed in the alkene hydroboration step (Scheme 3).

**Scheme 3:**
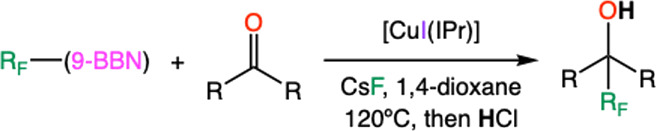
Synthesis of Fluoroalcohols by Copper-Catalyzed Addition of Fluorinated Alkylboranes to Symmetric Ketones

Subsequently, the system has been modified to achieve a better performance by changing the nature of both reactants and microkinetic simulations have been used to infer the activation barrier limit for getting fully working reactions. Finally, the electronic effects of both substrates on the overall reactivity of the system have been quantified by constructing a small reactant database that allowed connecting the activation barriers with the donor/acceptor properties of the starting materials by multilinear regression techniques.

## Computational Details

Theoretical calculations have been carried out using the GAUSSIAN16 package.^74^ The hybrid functional B3LYP has been employed for all the DFT calculations.^75–78^ In the geometry optimization procedures the Ahlrichs TZVP^79^ basis sets were used to describe all atoms except Cs and I, for which the def2-SVP (including ECP) basis sets were employed.^80–82^ Solvation energies in 1,4-dioxane were computed using the continuum dielectric solvation model (IEF-PCM)^83,84^ along with the SMD radii and nonelectrostatic terms.^85^ Dispersion effects were incorporated in the optimization process using the D3 method by Grimme.^86^ All minima and transition states were confirmed by vibrational analysis, which require zero and one imaginary frequencies, respectively. In all cases ultrafine integration grids were employed to carefully model the low-frequency vibrational modes (<100 cm^–^^1^) because of their significant contribution to entropy. These computational settings are denoted as scheme BS1.

Additionally, single-point calculations with larger basis sets were performed on the previously optimized geometries to improve the calculated Gibbs energies. The def2-TZVPPD^80–82,87,88^ basis sets were employed for all atoms while keeping the B3LYP functional together with the dispersion and solvation effects as in the optimization process (these computational settings are denoted as scheme BS2).

The computed Gibbs energies were adjusted to a standard state (1 M concentration in solution). At a given temperature, the Gibbs energy of a given compound was calculated using the rigid rotor/harmonic oscillator model, according to the following formula:

$$G_{T}^{0}=E_{\mathrm{BS}2}+H_{\mathrm{corr},\mathrm{BS}1}-TS_{\mathrm{BS}1}+RT\ln(C^{0}/C^{1\;\mathrm{atm}})$$ 1

In the given context, the symbols represent the following computed terms: *E*_BS2_ stands for the electronic energy, including solvent and dispersion effects, calculated using def2-TZVPPD basis sets (BS2); *H*_corr,BS1_ refers to the thermal correction to enthalpy, computed with the BS1 scheme; *S*_BS1_ represents the entropic correction derived from the BS1 scheme; *C* ^0^ denotes the standard reference state concentration (1 M), while *C* ^1 atm^ indicates the concentration of an ideal gas under standard pressure conditions at a specific temperature. For instance, at 120 °C, *C* ^1 atm^ is equal to 0.031 M, resulting in a final correction term of 2.71 kcal mol^–1^ per molecule. The energy terms of all the calculated species can be accessed in Table S1.

The reaction kinetics have been simulated using COPASI software.^89^ The rate constants for all forward and backward steps in the catalytic cycles were computed using DFT energy differences and the methodology described in the ESI. These rate constants were then fed into microkinetic models to generate transient concentrations of all species throughout the reaction. Kinetic simulations have been successfully employed to assess and support proposed reaction mechanisms.^90–94^

## Results and Discussion

### Characterization of the Reaction Mechanism

Initially, we examined the feasibility of the addition reaction of the fluorinated alkene to a symmetric ketone. To this end, we employed a simple symmetric ketone, and one of the fluorinated alkenes that had yielded good results in our previous work. Consequently, the reaction to be investigated is that between acetone and 4,4,4-trifluorobutene to yield 6,6,6-trifluoro-2-methyl-2-hexanol using [CuI­(IPr)] (IPr = 1,3-bis­(2,6-diisopropylphenyl)­imidazolinium) as catalyst (Scheme 4). As previously discussed, the hydroboration step has not been calculated, because prior results indicate that the addition of the 9-BBN dimer over the double bond of substituted alkenes produces mostly the anti-Markovnikov alkylborane derivative: CF_3_(CH_2_)_3_B­(C_8_H_14_) (R_F_-9-BBN), which is the one selected as starting material.

**Scheme 4:**
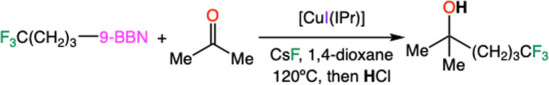
Computed Copper­(I)-Catalyzed Reaction between Acetone and 4,4,4-Trifluorobutene

According to the reaction mechanism proposed by Skrydstrup and Nielsen,^72^ and that computed by our group,^73^ the copper-catalyzed addition of the alkylborane onto a ketone follows the reaction sequence depicted in Figure 1.

**Figure 1:**
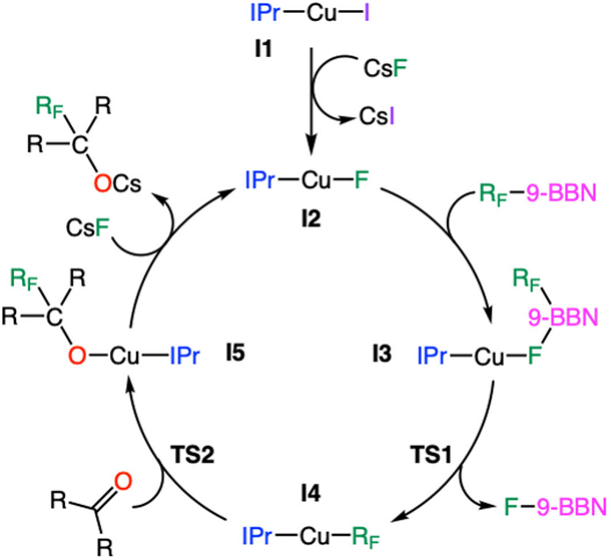
Proposed mechanism for the copper-catalyzed synthesis of fluorinated alcohols from alkylboranes (R_F_-9-BBN) and ketones (R_2_CO).

The proposed catalytic cycle provides the corresponding Gibbs energy profile shown in Figure 2. The reaction begins with the off-cycle halide replacement of the initial [CuI­(IPr)] complex (**I1**) with cesium fluoride to form the [CuF­(IPr)] intermediate (**I2**). The substitution of iodide by fluoride (**I1** → **I2**) is thermodynamically favorable with a relatively low decrease in Gibbs free energy of only 0.3 kcal mol^–1^. This process has been previously investigated and found to have a negligible energy barrier to proceed.^73^ In the second step (**I2** → **I3**), the copper-fluoride intermediate reacts with the organoborane (R_F_-9-BBN) to yield intermediate **I3**. The affinity of boron and fluoride to form B–F bonds is fundamental to facilitate the formation of this species. As in the previous stage, a low stabilization of intermediate **I3** is observed in terms of Gibbs energy, and this species is located at 3.0 kcal mol^–1^ below **I1**. Given that the fluoride ligand in **I2** is not sterically hindered, this addition stage is considered to be barrierless.^73^

**Figure 2:**
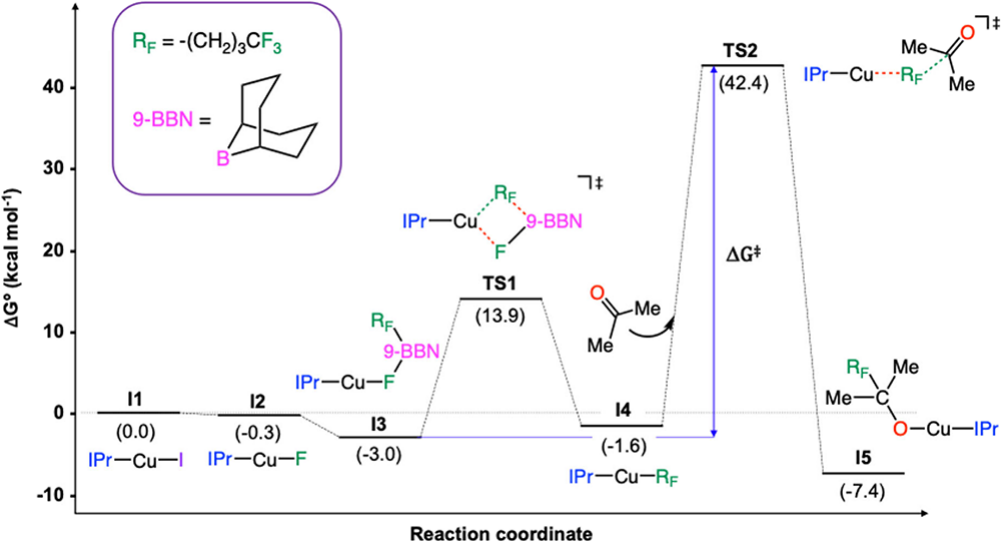
Gibbs energy profile of the copper-catalyzed addition of R_F_-9-BBN onto acetone.

The next step of the reaction is the transmetalation stage, in which the alkyl group of the borane is transferred from boron to copper (**I3** → **I4**). This process is governed by the corresponding transition state (**TS1**), which requires an energy investment of 16.9 kcal mol^–1^. In **TS1** (Figure 3a) the breaking Cu–F and C_Alk_–B bonds are clearly elongated from their values in intermediate **I3** (2.74 and 2.36 Å, respectively), while the forming Cu–C_Alk_ and B–F bonds clearly shorten (2.01 and 1.40 Å).

**Figure 3:**
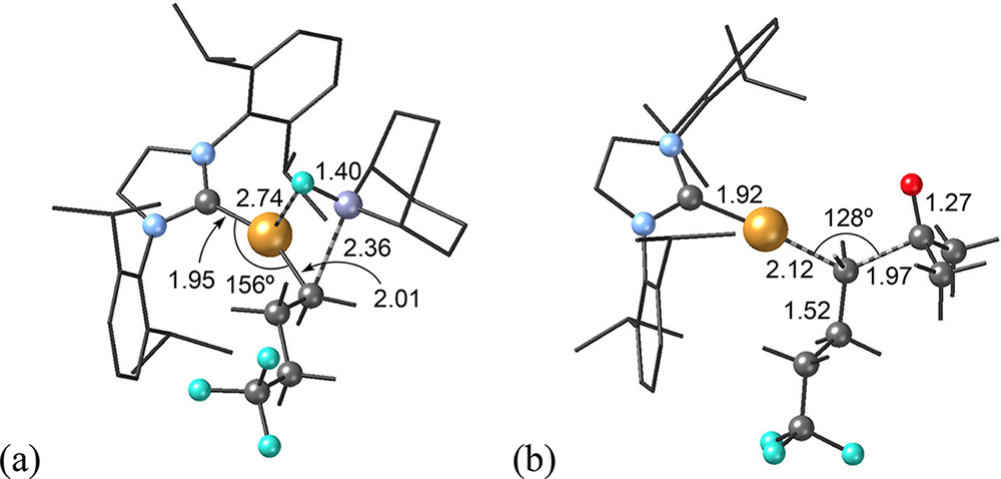
Calculated transmetalation (a, **TS1**) and C–C addition (b, **TS2**) transition states for the copper-catalyzed addition of R_F_-9-BBN onto acetone (distances in Å, angles in degrees; color code: C = gray, N = light blue, O = red, F = cyan, Cu = orange; the IPr ligand is represented as a wire and H atoms have been omitted for clarity).

Following the transmetalation the organometallic intermediate **I4** is formed. In this complex, the Cu–C_Alk_ bond distance is as short as 1.97 Å. The formation of this compound is almost thermoneutral and its computed overall Gibbs energy is −1.6 kcal mol^–1^. In the next step of the reaction, the copper-alkyl intermediate (**I4**) reacts with acetone, forming the corresponding copper-alkoxide derivative (**I4** → **I5**). This process is exergonic by 5.8 kcal mol^–1^, and the relative Gibbs energy of the latter intermediate is −7.4 kcal mol^–1^. This C–C coupling step consists of the S_N_-like nucleophilic attack of the coordinated alkyl ligand on the carbonyl group of the ketone, and it is controlled by its corresponding transition state (**TS2**). The structure of **TS2** (Figure 3b) shows a pentacoordinate carbon with the leaving and incoming groups at distances of 2.12 and 1.97 Å for Cu–C_Alk_ and C_Alk_–C_Ket_ respectively. The geometry around the alkylic carbon can be interpreted as highly distorted trigonal bipyramid with a Cu–C_Alk_–C_Ket_ angle of 128°. During **TS2** the C–O distance of free acetone elongates from 1.21 to 1.27 Å, leading to a certain degree of pyramidalization of the carbonyl group (347°). Energetically, the nucleophilic addition transition state (**TS2**) is found to be the highest energy species along the reaction coordinate (+42.4 kcal mol^–1^ above the starting materials). An alternative way of generating the C–C bond from **I4** has been also explored; in this case, the transition state has been calculated as a concerted 3-membered ring (Cu–C_Alk_–C_Ket_) species, like those computed in our previous study. However, this addition transition state showed a higher energy requirement than the linear **TS2** species and was consequently ruled out.

The final stage of the process consists of the reaction between **I5** and cesium fluoride to exchange of the alkoxide with fluoride; this replacement allows recovering the catalytic species **I2** and liberating the cesium alkoxide product, which can be afterward protonated to yield the desired fluoroalcohol. This step requires 6.5 kcal mol^–1^, producing a final Gibbs energy for the overall reaction of −0.6 kcal mol^–1^.

Therefore, the nucleophilic substitution emerges as the rate-limiting step of the whole process, and the overall Gibbs energy barrier (Δ*G* ^‡^), computed as the energy difference between **TS2** and **I3**, is 45.4 kcal mol^–1^. This large energy barrier indicates that the reaction is not likely to occur as proposed.

### Modification of the Catalytic System

As observed above, the thermodynamic and kinetic parameters of the proposed reaction to obtain fluoroalcohols indicate that the process cannot occur under the same reaction conditions as the alkene carboxylation process studied previously. The energy barrier for the alkene carboxylation process of 4,4,4-trifluorobutane-9-BBN with CO_2_ was determined to be 23.1 kcal mol^–1^;^73^ in contrast, the energy barrier for the addition of this substrate to acetone has nearly doubled. This discrepancy can be attributed to the higher electrophilic character of carbon dioxide, which results in a drastic reduction in the energy required for the attack of the alkyl group during **TS2** in the C–C bond formation process. In fact, the nature of this transition state, defined as the nucleophilic substitution of the coordinated alkyl substituent onto the carbonyl group of the ketone, should be expected to be favored by electron-rich alkyls and electron-poor ketones. Therefore, the feasibility of the reaction was explored to reverse the electronic demand of the starting materials. On one hand, the initial alkylborane was stripped of the fluoride substituents to provide an improved electron donor. On the other hand, since the final aim of this study is to provide a catalytic process to prepare fluoroalcohols, an electron-poor fluorinated ketone was used as the synthetic counterpart. Thus, the whole catalytic cycle was recomputed for the reaction between CH_3_(CH_2_)_3_B­(C_8_H_14_) (Bu-9-BBN) and hexafluoroacetone ((CF_3_)_2_CO) and compared to that calculated above. Figure 4 shows the Gibbs energy profiles for the copper-catalyzed reactions between R_F_-9-BBN + acetone and Bu-9-BBN + hexafluoroacetone. As may be observed, both energy profiles exhibit a parallel trend until the formation of the organometallic **I4** complex.

**Figure 4:**
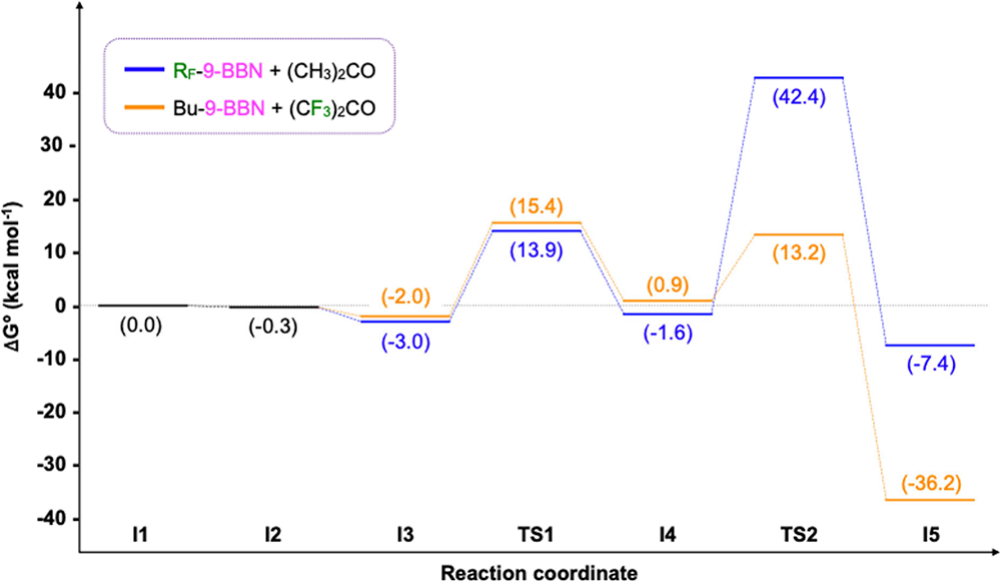
Gibbs energy profiles of the copper-catalyzed reactions between R_F_-9-BBN and acetone (blue), and Bu-9-BBN and hexafluoroacetone (orange).

The primary distinction between the two systems is observed in the nucleophilic substitution transition state (**TS2**). As previously hypothesized, the addition of the alkyl group to the electron-poor ketone results in a significantly reduced barrier, most likely due to the enhanced donor ability of the butyl group in the alkylborane and the more pronounced electrophilic nature of the carbonyl in hexafluoroacetone. The geometric arrangement in **TS2** is similar for both systems; the main differences are observed in the Cu–C_Alk_ and C_Alk_–C_Ket_ distances (Figures 3b and 5). In the case of reacting R_F_-9-BBN with acetone the Cu–C_Alk_ distance in **TS2** is quite longer than that found in its corresponding **I4** species (2.12 and 1.97 Å, respectively) while the C_Alk_–C_Ket_ distance is relatively short (1.97 Å). In contrast, for the reaction between Bu-9-BBN and hexafluoroacetone the inverse trend is observed in **TS2**: C_Alk_–C_Ket_ = 2.32 Å and Cu–C_Alk_ = 2.05 Å. These observations suggest that the Bu-9-BBN + (CF_3_)_2_CO reaction may involve an early transition state, while the R_F_-9-BBN + acetone system may exhibit a late transition state. This is consistent with the exergonic nature of their respective nucleophilic substitution stages.

**Figure 5:**
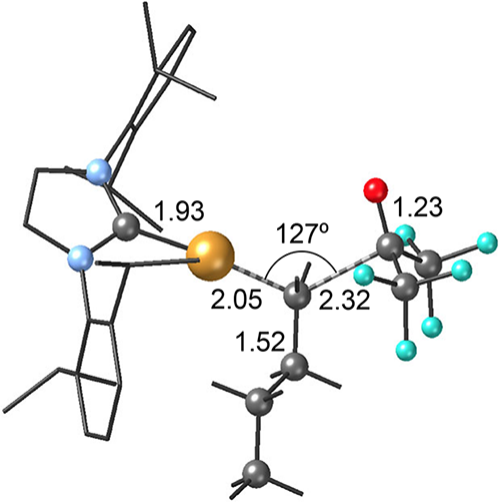
Calculated nucleophilic substitution transition state **(TS2**) for the copper-catalyzed addition of Bu-9-BBN onto hexafluoroacetone (distances in Å, angles in degrees; color code: C = gray, N = light blue, O = red, F = cyan, Cu = orange; the IPr ligand is represented as a wire and H atoms have been omitted for clarity).

The overall Gibbs energy barriers for both systems differ significantly. For the reaction of R_F_-9-BBN with acetone, as previously described, the barrier is calculated as the energy difference between **TS2** and **I3** (45.4 kcal mol^–1^). Conversely, in the Bu-9-BBN + hexafluoroacetone system, the energy barrier is as low as 17.4 kcal mol^–1^. Notably, this energy difference is observed between **I3** and **TS1**, indicating that the transmetalation of the nonfluorinated alkylborane is the most energy-demanding reaction stage.

The calculations for both catalytic systems indicate that the electronic effects of the substrates have a clear impact on the overall energy barrier of the reaction. However, the individual contribution of each reactant cannot be directly ascertained from the computed Gibbs energy profiles. To explore the influence of each substrate, several catalytic systems were computed in which the alkylborane and the symmetric ketone were modified with different functional groups (Figure 6). These calculations entail the combination of seven distinct alkylboranes with four different symmetric ketones. The seven alkylboranes contain different functional groups, covering a wide range of electronic inductive effects, in the farthest carbon to the borane end: R1-(CH_2_)_3_-9-BBN, where R1 = CH_3_, CF_3_, H_2_NCH_2_, MeOCH_2_, HOCH_2_, NCCH_2_, and O_2_NCH_2_. In the case of the symmetric ketones, different degrees of fluorination have been introduced in the methyl groups of acetone: (R2)_2_CO, with R2 = CH_3_, CH_2_F, CHF_2_ and CF_3_. This wide range of variations should allow a complete analysis of the studied reactivity, and help identifying the most relevant features of the substrates within the catalytic system. It should be noted that some of the products derived from this approach, primarily those resulting from acetone, do not generate fluoroalcohols. Nevertheless, these combinations of reactants facilitate completing the final analysis of the substrate effects on the reaction barriers.

**Figure 6:**
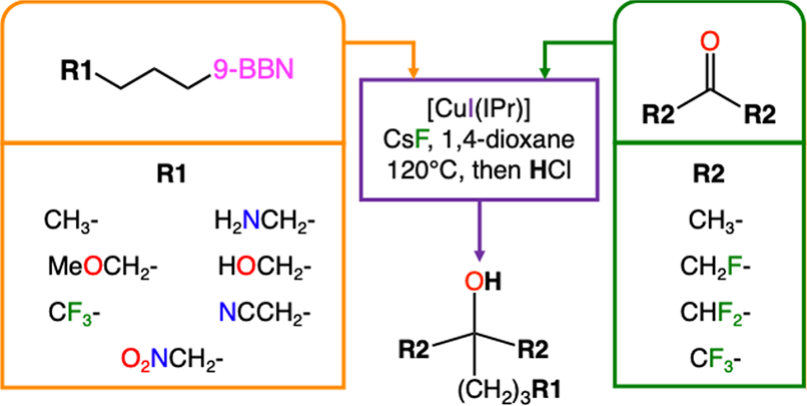
Combinations of substituted alkylboranes and symmetric ketones for the copper-catalyzed preparation of fluoroalcohols.

### Influence of the Substrates onto the Reaction Barrier

The reaction mechanism for all the possible substrate combinations (28 in total) was calculated and the activation barriers of all the systems have been identified (Figure 7 and Table S2). Most barriers are determined by the difference in energies between **TS2** and **I3**, indicating that the C_Alk_–C_Ket_ bond formation step is the slowest of the reaction. However, there are some exceptions, as indicated by the asterisk in Figure 7, in which the barrier is located between **TS1** and **I3**, corresponding to the transmetalation stage. It is noteworthy that these combinations are invariably observed when hexafluoroacetone is used as the ketone reactant. This phenomenon is probably due to the very strong electrophilicity of this substrate, which greatly facilitates the nucleophilic attack of the alkyl group onto the carbonyl.

**Figure 7:**
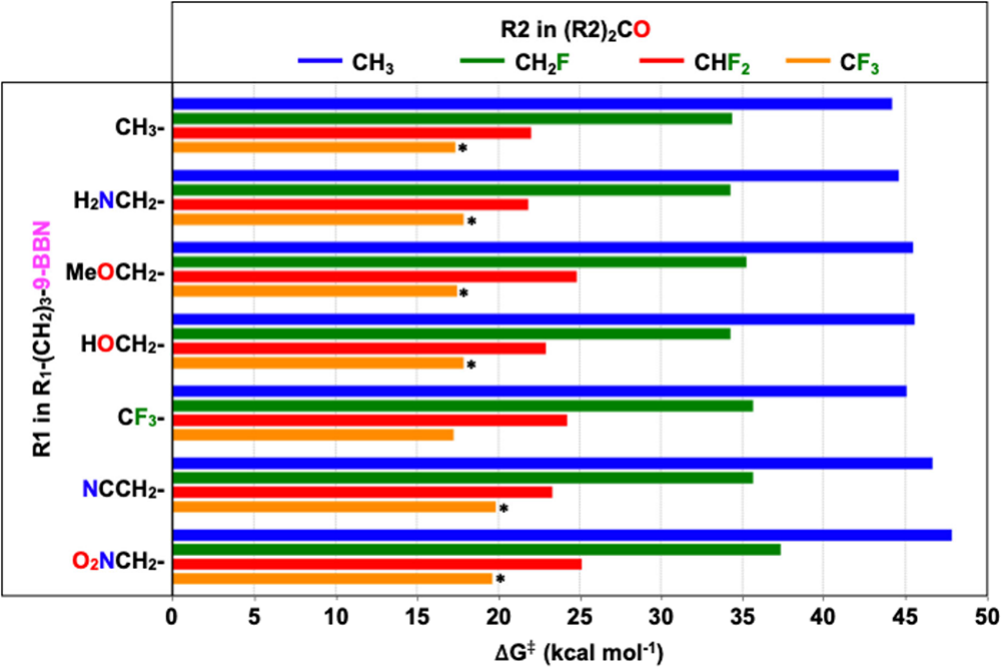
Calculated activation energies for the 28 possible combinations of alkylboranes and ketones in the copper-catalyzed synthesis of fluoroalcohols. The “*” symbol identifies the reactions with overall barriers corresponding to the transmetalation stage.

In general, it may be inferred that ketones exert a significantly greater influence on the calculated reaction barriers. Indeed, the barriers exhibit a range of approximately 28 kcal mol^–1^ when the symmetric ketones are varied (horizontal series in Figure 7), whereas when the alkylboranes are modified, the barriers display a narrow range of about 3 kcal mol^–1^ (vertical series in Figure 7). In addition, the calculated activation energies indicate that those reactions utilizing hexafluoroacetone (R2 = CF_3_) and 1,1,3,3-tetrafluoroacetone (R2 = CHF_2_) would be feasible at relatively mild conditions; on the other hand, the less fluorinated ketones would be very difficult to activate in the studied copper-catalyzed reactions.

As may be observed, the sequential replacement of hydrogens by fluorides in the methyl groups of acetone reduces the overall reaction barrier by roughly 10 kcal mol^–1^ for each substitution; the average barriers found for reactions using (CH_3_)_2_CO, (CH_2_F)_2_CO, (CHF_2_)_2_CO and (CF_3_)_2_CO are 45.9, 35.5, 23.6, and 18.3 kcal mol^–1^, respectively. This behavior is observed for all the reaction series (Figure 7). To illustrate this phenomenon, the Gibbs energy profiles of the combinations where CH_3_(CH_2_)_3_-9-BBN (Bu-9-BBN) reacts with the four ketones are shown in Figure 8. Obviously, in the series of reactions of Bu-9-BBN, the Gibbs energies of the species from **I1** to **I4** are the same because they depend only on the starting catalyst and the alkylborane. The effects of fluorination of acetone show their impact during the nucleophilic attack stage (**TS2**), which can be explained in terms of the electronic structure of the ketones. The fluorination of the α-carbon of acetone leads to an increase in the attraction of electron density, thereby enhancing the electrophilicity of the carbonyl group.

**Figure 8:**
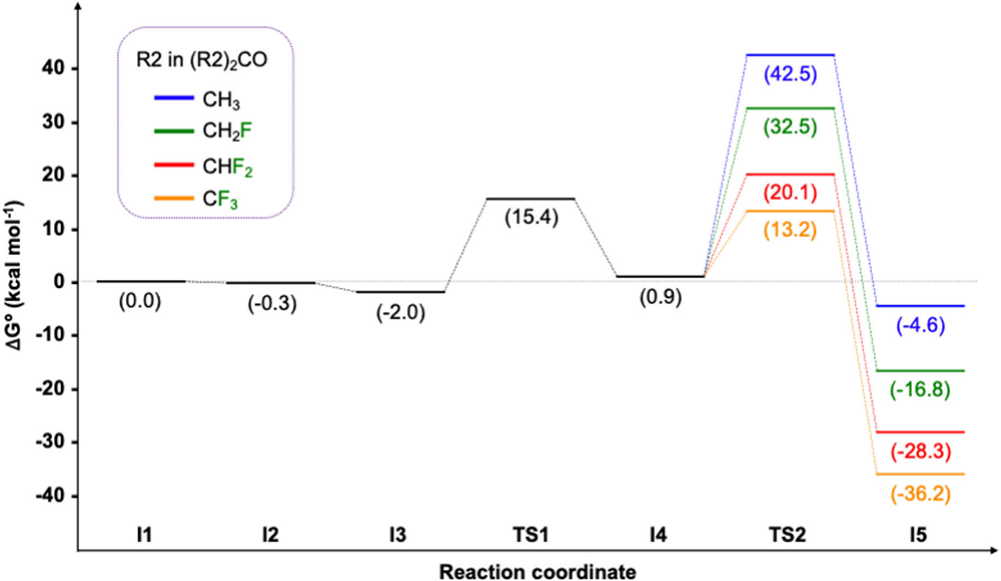
Gibbs energy profiles of the copper-catalyzed reactions between Bu-9-BBN and the four studied ketones.

In the case of hexafluoroacetone, the electrophilicity is so notable that the nucleophilic attack barrier (**TS2**) becomes lower than that of transmetalation (**TS1**). This may be related to the high reactivity of fluoroketones, which have become an important class of organics to synthesize new fluorinated pharmaceuticals due to their efficiency.^95–98^ The Gibbs energies for the formation of intermediate **I5**, follow a parallel behavior and can be located at about 50 kcal mol^–1^ below **TS2** in all cases. Therefore, it can be concluded that the fluorination of the ketone has a clear kinetic impact via decreasing the overall energy barrier, but also has a thermodynamic effect, which stabilizes species **I5.**

As mentioned before, the effect of the alkylboranes seems less relevant for the whole reactivity of the system, which is dominated by the identity of the ketone. However, a trend may be also observed for these reactants; typically, the barriers of alkylboranes containing electron-rich groups (e.g., CH_3_ and NH_2_) tend to be found in the lower range while reactants with electron-withdrawing groups, such as NO_2_ and CN, usually produce higher barriers. As an example, the reaction barriers of acetone with the seven alkylboranes are shown in Figure 9. As can be observed, the more electron-rich alkylboranes produce lower activation barriers, which may be related to their role as the nucleophilic counterpart of the reaction. In some cases, the calculated reaction barriers present deviations; for example, in this series the barrier for the alkylborane containing the trifluoromethyl group seems to be too low. Nevertheless, the calculated values are not far from those that should be obtained and are within the expected error for the DFT method, which is usually about 2 kcal mol^–1^. In any case, it seems that the alkylborane also plays a role in defining the overall reactivity of the system, and that may be related to its electronic properties.

**Figure 9:**
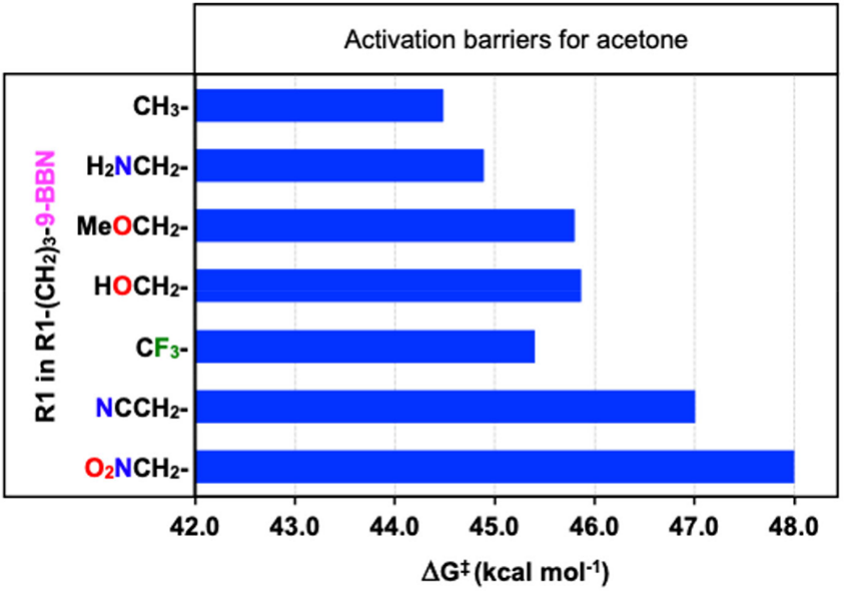
Calculated overall barriers for the copper-catalyzed reactions between acetone and different alkylboranes.

Thus, far, reaction barriers have been evaluated for various alkylborane and ketone substrate pairs; however, no quantitative predictions have been made regarding the functionality of these systems under the experimental reaction conditions. One way to evaluate the feasibility of the studied processes is to build microkinetic models, which estimate the time evolution of systems from Gibbs energies calculated with DFT. To this end, we constructed the microkinetic models for the reactions between Bu-9-BBN and the four ketones used above. The simulated kinetics indicate that the reactions with (CF_3_)_2_CO and (CHF_2_)_2_CO are very fast, and the reactions are complete in 1 and 22 s, respectively. On the other hand, the reactions with (CH_2_F)_2_CO and (CH_3_)_2_CO are very slow, with product yields of 2.4 and 0.0%, respectively, in 16-h runs. The kinetics data of these reactions can be found in the ESI. These results are in clear agreement with the calculated barriers and suggest that the reactions carried out with the most fluorinated ketones will produce very fast processes while the less fluorinated ketones will not provide functional catalytic systems. To get a more general vision of the relationship between the reaction barrier and the final product yield we have conducted a thorough exploration of the barrier influence on the performance of the reaction. To this purpose we have recomputed the microkinetic model of the Bu-9-BBN + (CHF_2_)_2_CO system manually fixing the height of **TS2** to produce integer values of the overall activation barrier between 23 and 35 kcal mol^–1^. These microkinetic models produce the time evolution of product yields shown in Figure 10; the activation barriers below 26 kcal mol^–1^ produce almost instantaneous reactions and, therefore, have not been included in this figure. As may be observed, the microkinetic modeling predicts that a reaction with an overall activation barrier around 29 kcal mol^–1^ (or lower) should produce quantitative conversions, and this value may be employed as a criterion to ascertain the feasibility of new calculated reactions. On the other hand, reactions with barriers higher than 29 kcal mol^–1^ should produce modest yields, which would decrease rapidly as the reaction barrier increases. This 29 kcal mol^–1^ limit has also been verified in the reactions between Bu-9-BBN and the other three ketones, which produce 99.7, 99.5 and 31.7% yields for (CF_3_)_2_CO, (CHF_2_)_2_CO and (CH_3_)_2_CO, respectively. The last yield is clearly affected by the poor stabilization of intermediate **I5**, which increases the reversibility of the C–C addition stage. This may be considered an artifact of the microkinetic modeling, in which the Gibbs energy of **TS2** has been lowered while all the others have been kept fixed at their original values. In principle, as observed in Figure 8, the Gibbs energies of **TS2** and **I5** are directly correlated, and therefore lowering **TS2** would entail also lowering **I5** by a similar amount, which has not been done in this barrier exploration. In any case, the 29 kcal mol^–1^ limit barrier seems a good estimation to determine the feasibility of the studied reactions.

**Figure 10:**
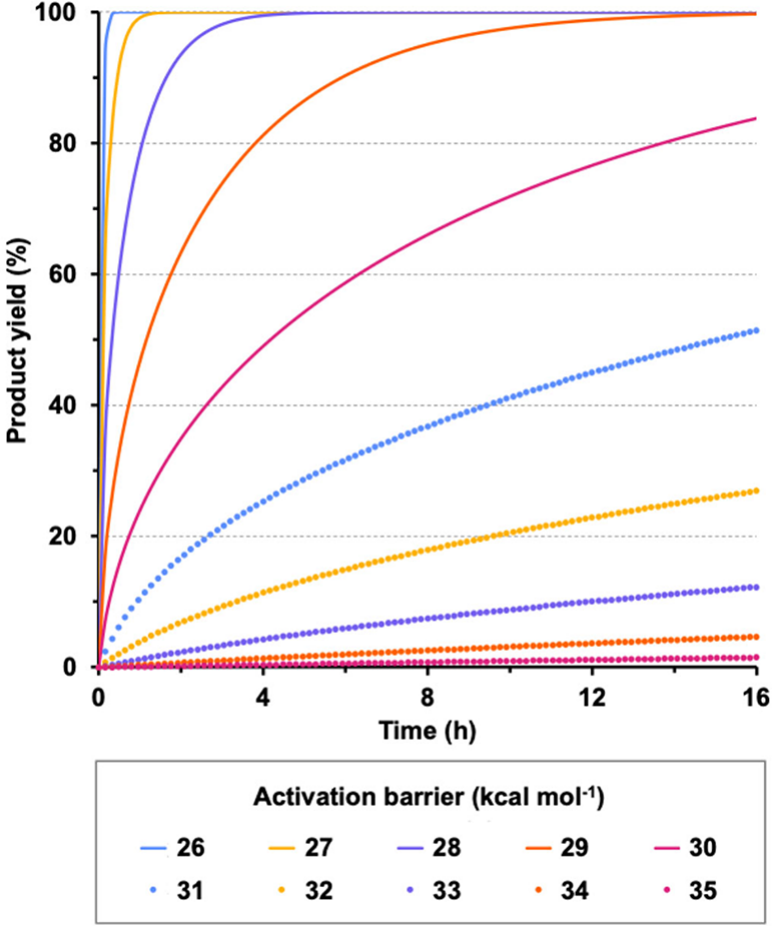
Time evolution of product yields for reactions between Bu-9-BBN + (CHF_2_)_2_CO with fixed activation barriers.

### Statistical Learning for Barrier Estimation

As stated above, the reaction barriers show a clear dependence on the electronic properties of both the symmetric ketone and the alkylborane substrates. An effective way to understand the impact of each reactant on the overall reactivity of the system is to construct a quantitative model based on multilinear regression techniques (MLR). The goal of this model is to quantitatively correlate the overall activation barrier for any combination of ketone and alkylborane with their electronic properties, codified in the shape of different numerical descriptors. These parameters can be atomic charges derived with different population schemes, frontier orbital (HOMO and LUMO) energies, hardness/softness, electrophilicity and nucleophilicity indexes, electronic chemical potentials,^99^ or other features found in literature, such as those of Hammett, Taft, Hansch, etc.^100,101^ The full list of calculated descriptors can be found in Tables S3 and S4.

Considering the calculated catalytic cycle, the overall reaction barrier seems to largely depend on the electrophilic character of the ketone and, to a lesser extent, on the electron donating ability of the alkylborane. Thus, multilinear regression models to predict the calculated Δ*G* ^‡^ values were constructed using the molecular descriptors derived from the starting reactants. In the case of the alkylboranes the parameters employed, related to their electron-donating properties, were the energy of the HOMO orbital (ϵ_HOMO_), the electronic chemical potential (μ), the Mulliken electronegativity (χ), the Pearson softness (*S*), the electrodonating power (ω^–^), the N and N’ nucleophilicity indexes, the Mulliken, CM5 ^102^ and NBO^103^ charges of the boron and the first carbon in the alkyl chain, and the electronic inductive parameter (σ_i_) of the remote functional group attached to the alkyl chain. On the other hand, the ketones are characterized by the following electron-accepting properties: the energy of the LUMO orbital (ϵ_LUMO_), the electronic chemical potential (μ), the Mulliken electronegativity (χ), the Pearson hardness (η), the electrophilicity index (ω), the electroaccepting power (ω^+^), and the Mulliken, CM5 ^102^ and NBO^103^ charges of the carbon atom in the carbonyl. The best correlation was found with the nucleophilicity index of the alkylborane (*N*’) and the LUMO energy of the ketone (ϵ_LUMO_) (Figure 11). Using the MLR equation the calculated activation energies could be reliably reproduced: *R* ^2^ = 0.992, rmse = 0.97 kcal mol^–1^, MAE = 0.84 kcal mol^–1^; Figure 11 shows the representation of the DFT vs MLR Δ*G* ^‡^ values.

**Figure 11:**
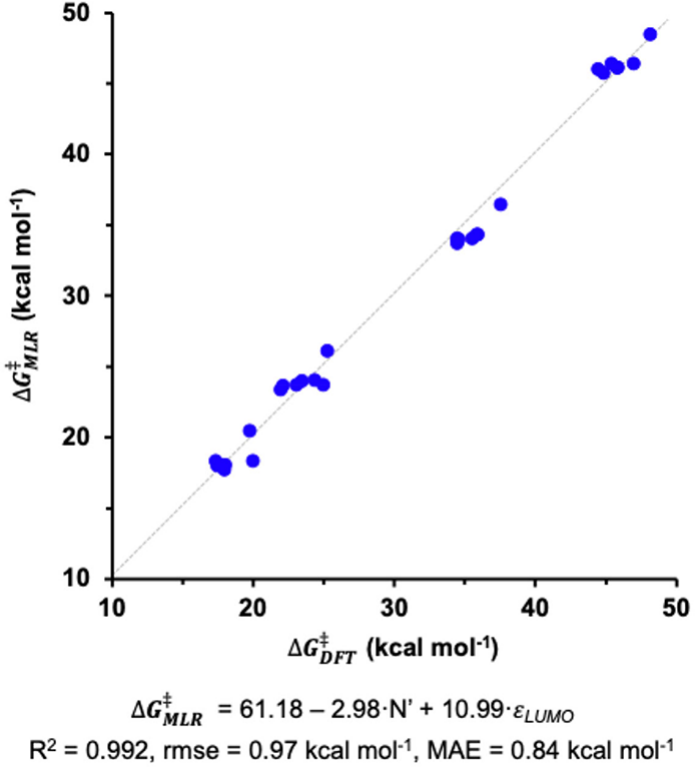
DFT vs. MLR Δ*G* ^‡^ for different ketone and alkylborane combinations.

As expected from previous observations, the linear regression coefficients indicate that highly electron-withdrawing groups in the alkylborane (higher *N*′ values) and more electrophilic ketones (lower ϵ_LUMO_ values) produce more favorable activation barriers and, therefore, improved catalytic systems. Furthermore, this approach allows to evaluate the relative importance of each descriptor within the correlation, quantifying its effect on the reaction barrier. This procedure consists of reconstructing the MLR with autoscaled descriptors, which produces an equivalent regression model with standardized regression coefficients that may be directly compared. Thus, the standardized MLR takes the shape:

$$\Delta G_{\mathrm{MLR}}^{⧧}=30.84-0.85\times N^{\prime }+10.69\times \varepsilon_{\mathrm{LUMO}}$$ 2

This expression indicates that the impact of the ketone on the overall activation barrier is ca. 13 times more important than that of the alkylborane. Since data availability is limited, the data set was not divided into training and test sets, which should provide an estimation of the predictive power of the constructed MLR model. However, a couple of cross-validation procedures were carried out. The leave-one-out cross-validation method produces *R* ^2^ = 0.990, rmse = 0.97 kcal mol^–1^ and MAE = 0.94 kcal mol^–1^, indicating the linear model is quite robust. This is confirmed by the 4-fold cross-validation procedure, which also generates very similar error values: rmse = 0.97 kcal mol^–1^, MAE = 0.94 kcal mol^–1^. The statistical parameters derived from the MLR models indicate that it would be possible to predict the activation barrier for similar systems without the need to calculate the entire catalytic cycle. The only parameters needed to estimate the barriers could be obtained from the calculation of the alkylborane and the symmetric ketone, as long as their characteristics remain close to those used to build the regression models. To support this statement, the activation barriers for different systems, not included in the original MLR development, have been computed with DFT and compared to those obtained using the expression shown in Figure 11 (Table 1). As may be observed, in most cases the MLR produces similar values to those obtained with the full DFT characterization, even for ketones bearing functional groups with increased steric effects such as CCl_3_ and *t*-Bu (Table 1, entries 1–5). The modification of the alkylborane chain (Table 1, entries 5–8) does not strongly affect the performance of the MLR and, again, the estimated values reproduce the DFT barriers in a relatively good agreement. Obviously, the quantitative agreement suffers when new and intrinsically different functional groups are included in the substrates. Nevertheless, the nature of the numerical descriptors employed, which derive from the electronic structure of the reactants, ensure, at least, a good qualitative agreement for new systems while they remain close to the original set of substrates employed in the development of the MLR.

**Table 1:** Comparison of Activation Barriers (in kcal mol^–1^) Calculated with DFT (Δ*G*_DFT_^‡^) and Obtained with the MLR (Δ*G*_MLR_^‡^) for Substrate Combinations Not Included in the Original Set of Reactants^a^

entry	R1	R2	**Δ** *G*_DFT_^‡^	**Δ** *G*_MLR_^‡^
1	CH_3_	2·*t*-Bu	48.8	46.4
2	CH_3_	*t*-Bu + CH_3_	46.9	46.6
3	CH_3_	*t*-Bu + CF_3_	30.4	33.2
4	CH_3_	CH_3_ + CF_3_	29.8	32.5
5	CH_3_	2·CCl_3_	19.4	19.8
6	HOCH_2_	2·CCl_3_	19.2	19.5
7	MeOCH_2_	2·CCl_3_	17.7	19.5
8	H_2_NCH_2_	2·CCl_3_	18.3	19.1
9	CH_3_	CF_3_ + CH_2_CN		23.8
10	CH_3_	CF_3_ + CH_2_NH_2_		25.6
11	CH_3_	CF_3_ + Ph		24.3
12	CH_3_	CF_3_ + *p*-NO_2_–C_6_H_4_		13.1
13	CH_3_	CF_3_ + *p*–Br-C_6_H_4_		22.3
14	CH_3_	CHF_2_ + CH_2_CN		26.1
15	CH_3_	CHF_2_ + CH_2_NH_2_		27.8
16	CH_3_	CHF_2_ + Ph		26.2
17	CH_3_	CHF_2_ + *p*-NO_2_–C_6_H_4_		14.5
18	CH_3_	CHF_2_ + *p*-Br–C_6_H_4_		24.1

a **R1** = R1 in R1-(CH_2_)_3_-9-BBN, **R2** = R2 in (R2)_2_CO.

Finally, new substrate combinations can be explored with the constructed MLR. For instance, new ketones bearing one trifluoromethyl or difluoromethyl group along with other substituents, which have been extracted from Reaxys, have been explored (Table 1, entries 9–18). It is important to note that some of these substrates have functional groups that can be further functionalized, i.e., CN, NH_2_, NO_2_ and Br, increasing the versatility of the prepared fluoroalcohols. As may be observed, all the additional ketones present barriers lower than 29 kcal mol^–1^, which should allow the reactions to proceed smoothly under the selected experimental conditions. As should be expected, the most electron poor ketones present the lowest activation barriers, for instance, both ketones bearing the phenyl ring with a *p*-NO_2_ substituent (Table 1, entries 12 and 17) show barriers below 15 kcal mol^–1^, making them very promising candidates.

## Conclusions

This computational study presents a plausible mechanism for synthesizing fluorinated alcohols from alkylboranes and ketones using a copper­(I) species as a catalyst. The proposed approach enhances the versatility of the original process by incorporating symmetric ketones as a substitute for carbon dioxide as the starting material, facilitating the synthesis of novel fluorinated compounds.

The calculated reaction mechanism indicates that, in most cases, the formation of the C–C bond between the alkyl group and the ketone acts as the rate-limiting step in the process. This stage demonstrates a clear dependence on the electronic nature of the organic substrates. Furthermore, computational analysis indicates that reaction barriers decrease significantly when ketones with electron-withdrawing substituents are used as reactants. Conversely, electron-rich alkylboranes exhibit a tendency to reduce the reaction barrier, although their impact is modest. Overall, the combination of electron-deficient ketones and electron-rich alkenes appears to be the most favorable configuration for carrying out the reaction.

The microkinetic modeling of the studied systems allows relating the computed activation barriers with the experimental feasibility of the reactions, showing that the most promising systems are those where the trifluoromethylated or difluoromethylated analogs of acetone are used. Furthermore, microkinetic analyses indicate that the activation barrier limit for constructing working reactions is approximately 29 kcal mol^–1^.

A comprehensive analysis using multilinear regression techniques contributes to a deeper understanding of the studied chemical transformation, providing a predictive tool for estimating reaction barriers, which can be further employed to predict the performance of alternative substrate combinations.

## Supplementary Material





## Data Availability

The data underlying this study are available in the published article and in its Supporting Information and openly available in the ioChem-BD database at 10.19061/iochem-bd-6-527.

## Notes

DFT calculated energy terms and lowest vibrational frequencies for all species; activation barriers for all of the studied systems; molecular descriptors for all of the alkylborane and ketone substrates; and microkinetic modeling procedures and related data (PDF)
Cartesian coordinates of the computed species (XYZ)
