# Supplementary material for: Computational Study of a Copper-Catalyzed Synthesis of Fluoroalcohols from Alkylboranes and Ketones
Source: J Org Chem. 2025 Sep 3;90(36):12591–601. doi: 10.1021/acs.joc.5c01174 (PMC12442092; doi:10.1021/acs.joc.5c01174)
Supplement: Supplementary file 1 [file jo5c01174_si_001.pdf]

Electronic supplementary information for:

Computational study of a copper-catalyzed synthesis of fluoroalcohols from alkylboranes and ketones

Francisco A. Gómez-Mudarra,[a,b] Gabriel Aullón,*[a,b] Jesús Jover*[,a,b]

[a] Secció de Química Inorgànica, Departament de Química Inorgànica i Orgànica, Universitat de Barcelona, Martí i Franquès 1-11, 08028, Barcelona, Spain.

[b] Institut de Química Teòrica i Computacional (IQTC-UB), Universitat de Barcelona, Martí i Franquès 1-11, 08028, Barcelona, Spain.

* Correspondence to Gabriel Aullón (gabriel.aullon@qi.ub.edu) or to Jesús Jover (jjovermo@ub.edu)

TABLE OF CONTENTS

**Table S1**: Energy terms and lowest vibrational frequencies for all the species involved in the catalytic cycles	S2
**Table S2**: Calculated activation barriers for all systems.	S7
**Table S3**: Molecular descriptors employed to describe the alkylboranes.	S8
**Table S4**: Molecular descriptors employed to describe the symmetric ketones	S9
**Table S5**: LUMO energies for additional ketone substrates	S10
Microkinetic modeling	S11
**Table S6**: Computed rate constants for all the studied reactions	S13
References	S14

Table S1. Energy terms (E_{BS2}, $H_{corr,BS1}$, TS_{BS1} and G^o, in Hartrees), for all the species involved in the catalytic cycles, and lowest vibrational frequency ($\tilde{\nu}$ in cm^{-1}).

Generic compounds	E_{BS2}	$H_{corr,BS1}$	TS_{BS1}	G^o	$\tilde{\nu}$
CsI	-318.0785	0.0033	0.0421	-318.1116	71.46
CsF	-120.1316	0.0034	0.0370	-120.1596	257.45
F-9BBN	-438.2483	0.2219	0.0576	-438.0784	60.12
$(CH_3)_2CO$	-193.2516	0.0888	0.0456	-193.2028	56.79
$(CH_2F)_2CO$	-391.7946	0.0762	0.0501	-391.7630	81.01
$(CHF_2)_2CO$	-590.3591	0.0623	0.0560	-590.3472	37.97
$(CF_3)_2CO$	-788.9376	0.0467	0.0598	-788.9452	36.60
(*t*-Bu)$_2$CO	-429.2347	0.2678	0.0663	-429.0289	37.64
(*t*-Bu)(CH_3)CO	-311.2475	0.1788	0.0561	-311.1205	38.95
(*t*-Bu)(CF_3)CO	-609.0970	0.1579	0.0628	-608.9975	42.89
$(CH_3)(CF_3)CO$	-491.1023	0.0690	0.0534	-491.0824	22.84
$(CCl_3)_2CO$	-2950.9406	0.0394	0.0682	-2950.9650	35.73
$(CF_3)(CH_2CN)CO$	-583.3653	0.0700	0.0579	-583.3488	35.59
$(CF_3)(CH_2NH_2)CO$	-510.3091	0.0879	0.0568	-510.2738	36.72
(CF_3)(Ph)CO	-682.9176	0.1255	0.0625	-682.8503	29.08
(CF_3)(*p*-NO_2-C_6H_4)CO	-887.5080	0.1302	0.0721	-887.4456	26.13
(CF_3)(*p*-Br-C_6H_4)CO	-3256.5008	0.1167	0.0683	-3256.4481	34.42
$(CHF_2)(CH_2CN)CO$	-484.0760	0.0777	0.0565	-484.0505	41.45
$(CHF_2)(CH_2NH_2)CO$	-411.0124	0.0957	0.0542	-410.9665	55.22
(CHF_2)(Ph)CO	-583.6264	0.1334	0.0606	-583.5493	30.71
(CHF_2)(*p*-NO_2-C_6H_4)CO	-788.2178	0.1381	0.0706	-788.1460	18.42
(CHF_2)(*p*-Br-C_6H_4)CO	-3157.2100	0.1246	0.0663	-3157.1473	42.46
CF_3-$(CH_2)_3$-9-BBN	-794.1022	0.3252	0.0817	-793.8532	21.72
CH_3-$(CH_2)_3$-9-BBN	-496.2284	0.3461	0.0742	-495.9509	23.56
H_2N-$(CH_2)_3$-9-BBN	-551.6026	0.3652	0.0783	-551.3101	25.65
OH-$(CH_2)_3$-9-BBN	-571.4807	0.3523	0.0784	-571.2012	23.42
MeO-$(CH_2)_3$-9-BBN	-610.7980	0.3816	0.0830	-610.4938	24.79
O_2N-$(CH_2)_3$-9-BBN	-700.8272	0.3523	0.0837	-700.5530	23.92
NC-$(CH_2)_3$-9-BBN	-588.5084	0.3471	0.0802	-588.2360	22.13

Common steps	E_{BS2}	$H_{corr,BS1}$	TS_{BS1}	G^o	$\tilde{\nu}$
**I1**	-3099.1040	0.6064	0.1346	-3098.6266	19.77
**I2**	-2901.1573	0.6067	0.1300	-2900.6750	21.98

CF_3-$(CH_2)_3$-9-BBN		E_{BS2}	$H_{corr,BS1}$	TS_{BS1}	G^o	$\tilde{\nu}$
	**I3**	-3695.2975	0.9358	0.1765	-3694.5326	18.83
	**TS1**	-3695.2669	0.9341	0.1784	-3694.5057	-147.80
	**I4**	-3257.0124	0.7096	0.1548	-3256.4520	10.08
**$(CH_3)_2CO$**	**TS2**	-3450.2229	0.8001	0.1675	-3449.5847	-449.04
	**I5**	-3450.3070	0.8044	0.1669	-3449.6639	11.39
	**Cs_product**	-669.2621	0.1991	0.0808	-669.1382	18.15
**$(CH_2F)_2CO$**	**TS2**	-3648.7813	0.7879	0.1720	-3648.1599	-284.52
	**I5**	-3648.8680	0.7921	0.1714	-3648.2418	14.70
	**Cs_product**	-867.8346	0.1868	0.0858	-867.7280	21.56
**$(CHF_2)_2CO$**	**TS2**	-3847.3641	0.7740	0.1781	-3845.7626	-428.43
	**I5**	-3845.4518	0.7781	0.1749	-3846.8430	18.39
	**Cs_product**	-1066.4205	0.1727	0.0924	-1066.3346	12.77
**$(CF_3)_2CO$**	**TS2**	-4045.9550	0.7585	0.1808	-4045.3717	-373.86
	**I5**	-4046.0418	0.7622	0.1800	-4045.4541	16.12
	**Cs_product**	-1265.0124	0.1570	0.0961	-1264.9460	18.42

CH_3-$(CH_2)_3$-9-BBN		E_{BS2}	$H_{corr,BS1}$	TS_{BS1}	G^o	$\tilde{\nu}$
	**I3**	-3397.4199	0.9563	0.1707	-3396.6288	21.14
	**TS1**	-3397.3898	0.9549	0.1716	-3396.6010	-133.14
	**I4**	-2959.1343	0.7304	0.1475	-2958.5458	14.34
**$(CH_3)_2CO$**	**TS2**	-3152.3472	0.8208	0.1615	-3151.6823	-451.92
	**I5**	-3152.4301	0.8255	0.1584	-3151.7573	20.34
	**Cs_product**	-371.3856	0.2200	0.0731	-371.2331	25.42
**$(CH_2F)_2CO$**	**TS2**	-3350.9075	0.8086	0.1650	-3350.2583	-298.47
	**I5**	-3350.9923	0.8133	0.1635	-3350.3369	14.89
	**Cs_product**	-569.9591	0.2077	0.0781	-569.8240	27.31
**$(CHF_2)_2CO$**	**TS2**	-3549.4893	0.7945	0.1731	-3548.8623	-429.05
	**I5**	-3549.5769	0.7992	0.1674	-3548.9395	19.05
	**Cs_product**	-768.5451	0.1936	0.0846	-768.4305	19.07
**$(CF_3)_2CO$**	**TS2**	-3748.0800	0.7790	0.1759	-3747.4712	-334.08
	**I5**	-3748.1674	0.7833	0.1715	-3747.5500	15.50
	**Cs_product**	-967.1380	0.1779	0.0886	-967.0431	13.26
**$(t\text{-Bu})_2CO$**	**TS2**	-3388.3234	0.9997	0.1793	-3387.4987	-311.87
**$(t\text{-Bu})(CH_3)CO$**	**TS2**	-3270.3414	0.9100	0.1663	-3269.5934	-472.99
**$(t\text{-Bu})(CF_3)CO$**	**TS2**	-3568.2128	0.8899	0.1780	-3567.4966	-372.75
**$(CH_3)(CF_3)CO$**	**TS2**	-3450.2220	0.8012	0.1660	-3449.5825	-438.17
**$(CCl_3)_2CO$**	**TS2**	-5910.0730	0.7716	0.1839	-5909.4810	-375.97

H_2N-$(CH_2)_3$-9-BBN		E_{BS2}	$H_{corr,BS1}$	TS_{BS1}	G^o	$\tilde{\nu}$
	**I3**	-3452.7943	0.9754	0.1759	-3451.9892	15.35
	**TS1**	-3452.7647	0.9742	0.1757	-3451.9606	-116.69
	**I4**	-3014.5086	0.7495	0.1518	-3013.9053	13.46
**$(CH_3)_2CO$**	**TS2**	-3207.7236	0.8406	0.1647	-3207.0421	-435.38
	**I5**	-3207.8047	0.8448	0.1615	-3207.1159	19.83
	**Cs_product**	-426.7601	0.2390	0.0774	-426.5930	19.16
**$(CH_2F)_2CO$**	**TS2**	-3406.2833	0.8283	0.1695	-3405.6189	-279.00
	**I5**	-3406.3708	0.8325	0.1678	-3405.7005	15.78
	**Cs_product**	-625.3333	0.2268	0.0927	-625.1937	23.21
**$(CHF_2)_2CO$**	**TS2**	-3604.8656	0.8139	0.1769	-3604.2231	-417.65
	**I5**	-3604.9514	0.8183	0.1720	-3604.2994	16.30
	**Cs_product**	-823.9192	0.2126	0.0889	-823.7899	19.82
**$(CF_3)_2CO$**	**TS2**	-3803.4564	0.7983	0.1771	-3802.8296	-349.38
	**I5**	-3803.5421	0.8025	0.1765	-3802.9105	11.42
	**Cs_product**	-1022.5122	0.1970	0.0927	-1022.4024	19.26
**$(CCl_3)_2CO$**	**TS2**	-5985.3252	0.7776	0.1890	-5984.7323	-384.43

HO-$(CH_2)_3$-9-BBN		E_{BS2}	$H_{corr,BS1}$	TS_{BS1}	G^o	$\tilde{\nu}$
	**I3**	-3472.6727	0.9625	0.1751	-3471.8798	17.08
	**TS1**	-3472.6428	0.9613	0.1751	-3471.8510	-118.24
	**I4**	-3034.3870	0.7367	0.1518	-3033.7966	11.75
**$(CH_3)_2CO$**	**TS2**	-3227.5993	0.8272	0.1646	-3226.9311	-452.29
	**I5**	-3227.6839	0.8320	0.1611	-3227.0074	21.05
	**Cs_product**	-446.6383	0.2262	0.0776	-446.4841	19.05
**$(CH_2F)_2CO$**	**TS2**	-3426.1590	0.8149	0.1708	-3425.5094	-289.40
	**I5**	-3426.2498	0.8197	0.1673	-3425.5919	15.69
	**Cs_product**	-645.2115	0.2139	0.0826	-645.0746	24.21
**$(CHF_2)_2CO$**	**TS2**	-3624.7417	0.8008	0.1765	-3624.1118	-413.33
	**I5**	-3624.8307	0.8053	0.1728	-3624.1926	13.33
	**Cs_product**	-843.7974	0.1998	0.0888	-843.6808	15.30
**$(CF_3)_2CO$**	**TS2**	-3823.3319	0.7853	0.1786	-3822.7197	-359.63
	**I5**	-3823.4208	0.7897	0.1745	-3822.8000	12.64
	**Cs_product**	-1042.3904	0.1841	0.0930	-1042.2938	12.32
**$(CCl_3)_2CO$**	**TS2**	-5985.3252	0.7776	0.1890	-5984.7323	-385.10

MeO-$(CH_2)_3$-9-BBN		E_{BS2}	$H_{corr,BS1}$	TS_{BS1}	G^o	$\tilde{\nu}$
	**I3**	-3511.9904	0.9919	0.1798	-3511.1728	15.73
	**TS1**	-3511.9603	0.9906	0.1806	-3511.1448	-115.61
	**I4**	-3073.7044	0.7660	0.1564	-3073.0893	10.09
**$(CH_3)_2CO$**	**TS2**	-3266.9178	0.8565	0.1686	-3266.2243	-437.08
	**I5**	-3267.0006	0.8611	0.1667	-3266.3006	15.61
	**Cs_product**	-485.9556	0.2554	0.0828	-485.7774	15.56
**$(CH_2F)_2CO$**	**TS2**	-3465.4775	0.8444	0.1732	-3464.8007	-285.39
	**I5**	-3465.5650	0.8491	0.1715	-3464.8818	17.31
	**Cs_product**	-684.5288	0.2432	0.0875	-684.3676	20.49
**$(CHF_2)_2CO$**	**TS2**	-3664.0600	0.8304	0.1777	-3663.4018	-423.32
	**I5**	-3664.1473	0.8346	0.1777	-3663.4849	12.17
	**Cs_product**	-883.1148	0.2291	0.0935	-882.9736	13.60
**$(CF_3)_2CO$**	**TS2**	-3862.6498	0.8147	0.1831	-3862.0127	-354.42
	**I5**	-3862.7376	0.8189	0.1803	-3862.0934	12.34
	**Cs_product**	-1081.7076	0.2134	0.0967	-1081.5853	19.24
**$(CCl_3)_2CO$**	**TS2**	-6024.6429	0.8070	0.1956	-6024.0273	-384.15

O_2N-$(CH_2)_3$-9-BBN		E_{BS2}	$H_{corr,BS1}$	TS_{BS1}	G^o	$\tilde{\nu}$
	**I3**	-3602.0224	0.9625	0.1820	-3601.2364	10.46
	**TS1**	-3601.9929	0.9614	0.1789	-3601.2048	-107.99
	**I4**	-3163.7373	0.7367	0.1580	-3163.1530	9.79
**$(CH_3)_2CO$**	**TS2**	-3356.9502	0.8275	0.1669	-3356.2840	-405.91
	**I5**	-3357.0314	0.8321	0.1653	-3356.3591	16.78
	**Cs_product**	-575.9866	0.2261	0.0833	-575.8382	17.17
**$(CH_2F)_2CO$**	**TS2**	-3555.5092	0.8151	0.1726	-3554.8611	-250.65
	**I5**	-3555.5927	0.8199	0.1692	-3554.9365	19.55
	**Cs_product**	-774.5592	0.2138	0.0894	-774.4293	15.93
**$(CHF_2)_2CO$**	**TS2**	-3754.0923	0.8010	0.1792	-3753.4650	-402.43
	**I5**	-3754.1767	0.8059	0.1755	-3753.5408	16.49
	**Cs_product**	-973.1449	0.1997	0.0943	-973.0339	21.60
**$(CF_3)_2CO$**	**TS2**	-3952.6823	0.7856	0.1808	-3952.0719	-359.44
	**I5**	-3952.7673	0.7900	0.1810	-3952.1528	6.07
	**Cs_product**	-1171.7376	0.1840	0.0993	-1171.6473	13.63

NC-(CH$_2$)$_3$-9-BBN		E_{BS2}	$H_{corr,BS1}$	TS_{BS1}	G^o	$\tilde{\nu}$
	**I3**	-3489.7027	0.9573	0.1778	-3488.9176	9.84
	**TS1**	-3489.6730	0.9564	0.1747	-3488.8857	-119.85
	**I4**	-3051.4176	0.7315	0.1539	-3050.8343	10.35
**(CH$_3$)$_2$CO**	**TS2**	-3244.6307	0.8224	0.1644	-3243.9671	-412.68
	**I5**	-3244.7124	0.8264	0.1648	-3244.0452	15.64
	**Cs_product**	-463.6673	0.2210	0.0796	-463.5203	16.54
**(CH$_2$F)$_2$CO**	**TS2**	-3443.1898	0.8102	0.1710	-3442.5450	-269.18
	**I5**	-3443.2735	0.8143	0.1709	-3442.6246	13.93
	**Cs_product**	-662.2402	0.2087	0.0849	-662.1109	18.59
**(CHF$_2$)$_2$CO**	**TS2**	-3641.7729	0.7959	0.1775	-3641.1490	-409.55
	**I5**	-3641.8599	0.8001	0.1712	-3641.2254	19.57
	**Cs_product**	-860.8255	0.1946	0.0921	-860.7174	13.87
**(CF$_3$)$_2$CO**	**TS2**	-3840.3631	0.7804	0.1786	-3839.7556	-360.03
	**I5**	-3840.4481	0.7843	0.1771	-3839.8353	17.09
	**Cs_product**	-1059.4188	0.1789	0.0954	-1059.3297	12.37

Table S2. Calculated activation barriers for all systems (in kcal mol^{-1}).

	R2 in $(R2)_2CO$			
**R1 in R1-$(CH_2)_3$-9-BBN**	**CH_3**	**CH_2F**	**CHF_2**	**CF_3**
**CH_3**	44.5	34.6	22.2	17.5
**NH_2**	44.9	34.5	22.0	18.0
**OMe**	45.8	35.5	25.0	17.6
**OH**	45.9	34.5	23.1	18.0
**CF_3**	45.4	35.9	24.4	17.4
**CN**	47.0	35.9	23.5	20.0
**NO_2**	48.2	37.6	25.3	19.8

Table S3. Molecular descriptors employed to describe the alkylboranes.

	R1 in R1-$(CH_2)_3$-9-BBN						
	**NO_2**	**CN**	**CF_3**	**CH_3**	**OH**	**MeO**	**NH_2**
ε_{HOMO}	-6.9675	-6.9520	-6.9558	-6.8061	-6.8380	-6.8352	-6.5577
ε_{LUMO}	-2.1576	-0.4612	-0.4686	-0.3108	-0.3388	-0.3363	-0.3184
$\varepsilon_{\text{H-L}}$	4.8099	6.4907	6.4872	6.4954	6.4992	6.4989	6.2393
μ	-4.5625	-3.7066	-3.7122	-3.5584	-3.5884	-3.5858	-3.4380
χ	4.5625	3.7066	3.7122	3.5584	3.5884	3.5858	3.4380
η	2.4049	3.2454	3.2436	3.2477	3.2496	3.2494	3.1197
S	0.4158	0.3081	0.3083	0.3079	0.3077	0.3077	0.3205
ω	4.3279	2.1167	2.1242	1.9495	1.9812	1.9785	1.8944
ω^+	0.4839	0.0164	0.0169	0.0074	0.0088	0.0087	0.0081
ω^-	5.0465	3.7230	3.7291	3.5659	3.5972	3.5945	3.4461
N’	1.9816	2.6860	2.6816	2.8044	2.7799	2.7820	2.9018
N	0.7091	0.7246	0.7208	0.8705	0.8387	0.8414	1.1189
σ_i	0.76	0.53	0.42	0.00	0.29	0.27	0.12
$C_{Mulliken}$	-0.4594	-0.4692	-0.4022	-0.3662	-0.4225	-0.4259	-0.4056
C_{CM5}	-0.1510	-0.1520	-0.1504	-0.1546	-0.1531	-0.1531	-0.1539
C_{NBO}	-0.8139	-0.8150	-0.8136	-0.8179	-0.8155	-0.8152	-0.8162
$B_{Mulliken}$	-0.1815	-0.1710	-0.1679	-0.1174	-0.1369	-0.1305	-0.1371
B_{CM5}	-0.0893	-0.0895	-0.0887	-0.0904	-0.0903	-0.0903	-0.0905
B_{NBO}	1.0693	1.0699	1.0702	1.0703	1.0692	1.0692	1.0694

Descriptor list: Energy of the HOMO orbital (ε_{HOMO}), Energy of the LUMO orbital (ε_{LUMO}), HOMO-LUMO energy gap ($\varepsilon_{\text{H-L}}$), Electronic chemical potential (μ), Mulliken’s electronegativity (χ), Pearson’s hardness (η), Pearson’s softness (S), Electrophilicity Index (ω), Electroaccepting power (ω^+), Electrodonating power (ω^-), Nucleophilicity index (N’), relative Nucleophilicity index (N), all in eV. Electronic inductive parameter (σ_i), charges of the first C atom in the alkyl chain: Mulliken ($C_{Mulliken}$), CM5 (C_{CM5}), NBO (C_{NBO}), and charges of the B atom: Mulliken ($B_{Mulliken}$), CM5 (B_{CM5}), NBO (B_{NBO}).

Table S4. Molecular descriptors employed to describe the symmetric ketones

	R2 in $(R2)_2CO$			
	CH_3	CH_2F	CHF_2	CF_3
ε_{HOMO}	-6.9454	-7.6948	-8.3425	-9.1109
ε_{LUMO}	-0.6177	-1.7135	-2.6537	-3.1685
$\varepsilon_{\text{H-L}}$	6.3277	5.9813	5.6888	5.9424
μ	-3.7816	-4.7042	-5.4981	-6.1397
χ	3.7816	4.7042	5.4981	6.1397
η	3.1639	2.9907	2.8444	2.9712
S	0.3161	0.3344	0.3516	0.3366
ω	2.2599	3.6997	5.3137	6.3435
ω^+	0.0301	0.2454	0.6189	0.8447
ω^-	3.8117	4.9496	6.117	6.9844
N’	2.6235	2.0204	1.6348	1.4318
N	0.7312	-0.0182	-0.6659	-1.4343
$C_{Mulliken}$	0.6136	0.2772	0.2485	-0.0389
C_{CM5}	0.1859	0.1943	0.1958	0.2006
C_{NBO}	0.5903	0.5119	0.4523	0.4042

Descriptor list: Energy of the HOMO orbital (ε_{HOMO}), Energy of the LUMO orbital (ε_{LUMO}), HOMO-LUMO energy gap ($\varepsilon_{\text{H-L}}$), Electronic chemical potential (μ), Mulliken’s electronegativity (χ), Pearson’s hardness (η), Pearson’s softness (S), Electrophilicity Index (ω), Electroaccepting power(ω^+), Electrodonating power (ω^-), Nucleophilicity index (N’), relative Nucleophilicity index (N), all in eV. Charges of the C atom in the carbonyl: Mulliken ($C_{Mulliken}$), CM5 (C_{CM5}), NBO (C_{NBO}).

Table S5. LUMO energies (ε_{LUMO}, in eV) for additional ketone substrates.

**Ketone**	ε_{LUMO}
$(t\text{-Bu})_2CO$	-0.5818
$(t\text{-Bu})(CH_3)CO$	-0.5646
$(t\text{-Bu})(CF_3)CO$	-1.7889
$(CH_3)(CF_3)CO$	-1.8512
$(CCl_3)_2CO$	-3.0414
$(CF_3)(CH_2CN)CO$	-2.6384
$(CF_3)(CH_2NH_2)CO$	-2.4776
$(CF_3)(Ph)CO$	-2.5913
$(CF_3)(p\text{-}NO_2\text{-}C_6H_4)CO$	-3.6175
$(CF_3)(p\text{-Br-}C_6H_4)CO$	-2.7796
$(CHF_2)(CH_2CN)CO$	-2.4278
$(CHF_2)(CH_2NH_2)CO$	-2.2741
$(CHF_2)(Ph)CO$	-2.4180
$(CHF_2)(p\text{-}NO_2\text{-}C_6H_4)CO$	-3.4874
$(CHF_2)(p\text{-Br-}C_6H_4)CO$	-2.6104

Microkinetic modeling

Methodology

The microkinetic modeling, which permits the calculation of the transient concentrations of all the species during the reaction, has been constructed employing the COPASI[1] program. In all cases the the simple mass action law is used. The forward and backward rate constants for each reaction stage have been computed from the Gibbs energy differences within the reaction pathways. In these models all the steps are considered to be reversible even though the energy difference between some of them would point to a non-reversible behavior. In practice, this assumption does not produce significant differences in the calculated kinetics profiles. In all cases the temperature (120ºC), reaction time (16 hours) and initial concentrations of all the species in the model are those used in the experiments, *i.e.* [CuI(IPr)] = 0.01 M, [Alkylborane] = 0.2 M, [Ketone] = 0.4 M, and [CsF] = 0.6 M, while the concentration of all the other species has been set to zero. The computed rate constants for all the studied processes can be found in Table S5.

Rate constant calculations

Reactions of type $A + B \leftrightharpoons C$ (or its opposite), which proceed without a clear energy barrier on the potential energy surface, *i.e.* the association and dissociation steps, are considered diffusion-controlled elemental stages. Therefore, the rate constant in solution for the associative direction is calculated by the the Stokes-Einstein equation within the von Smoluchowski formulation: [2]

$$k_{diff} = 8k_B T N_A \cdot 10^3 / 3\eta \quad (M^{-1}s^{-1})$$

where k_B is the Boltzmann constant, T is the temperature, N_A is Avogadro's number and η is the solvent viscosity at that temperature. It must be noted that this expression is used for the association reaction, which may correspond either to the forward (k_f) or backward (k_b) rate constant of the process. Thus, once k_f or k_b is obtained, its reversal is easily computed through the formulation of the thermodynamic equilibrium constant K and the computed Gibbs energy difference between the intermediates involved ($\Delta G°$):

$$\Delta G° = -RTlnK$$

$$K = \exp\left(-\Delta G°/RT\right)$$

$$K = k_f/k_b$$

For example, in the barrierless reaction $A + B \leftrightharpoons C$, the forward rate constant k_f is equal to k_{diff}. The equilibrium constant K is calculated placing the Gibbs free energy in the expression above and, consequently, $k_b = k_f/K$. In the case of 1,4-dioxane, the viscosity at 120ºC could not be found in literature and it had to be estimated with a third-degree polynomial fit of the experimental data,[3] Figure S1. Using this polynomial fit, the viscosity of 1,4-dioxane at 120ºC (393.15 K) was estimated to be 0.437 mPa·s, yielding a diffusion-controlled rate constant k_{diff} of $1.993 \cdot 10^{10}$ $M^{-1}s^{-1}$.

T (K)	η (mPa·s)
293.15	1.313[3b]
298.15	1.177[3a]
303.15	1.101[3b]
308.15	1.014[3b]
313.15	0.943[3b]
323.15	0.787[3a]
348.15	0.569[3a]
393.15	0.437

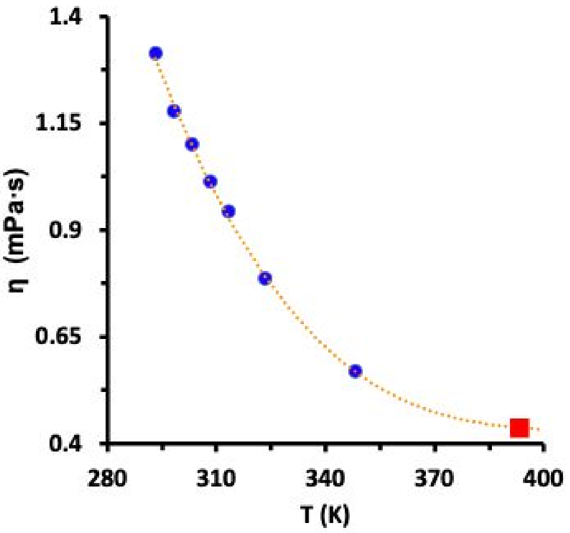

$$\eta = -0.0000005567 \cdot T^3 + 0.0006802426 \cdot T^2 - 0.2774517185 \cdot T + 38.2036404895$$
$$R^2 = 0.998$$

Figure S1. Experimental viscosity (η) data of 1,4-dioxane at different temperatures, third-degree polynomial fit of the data: equation (bottom) and graphical representation (right). The expected viscosity of 1,4-dioxane at the desired temperature (393.15 K) is shown at the bottom of the table and as a red square in the graph. The experimental values for the polynomial fit have been extracted from references 3a and 3b.

On the other hand, the rate constants of all the steps governed by a transition state are computed with the Eyring-Polanyi equation:[4]

$$k = \frac{k_B T}{h} \exp\left(-\Delta G^{\ddagger}/RT\right)$$

where k_B is the Boltzmann constant, T is the temperature, h is the Planck constant, $\Delta G^{\ddagger}$ is the activation Gibbs energy and R is the gas constant. The forward (k_f) and backward (k_b) rate constants are calculated independently using their own activation energies. For instance, in a reaction like $A \leftrightharpoons B$ with a transition state TS_{AB} connecting both species, $\Delta G^{\ddagger}_{forward} = G^{\circ}_{TSAB} - G^{\circ}_{A}$ while $\Delta G^{\ddagger}_{backward} = G^{\circ}_{TSAB} - G^{\circ}_{B}$. Besides, the forward and backward rate constants of all the bimolecular substitution stages, *i.e.* $A + B \leftrightharpoons C + D$, without a clear transition state have been also computed employing the Eyring-Polanyi equation. These steps are considered fast processes with an associated fictional 2 kcal mol^{-1} barrier in the endergonic direction of the reversible step. Note that all the bimolecular reactions, where the rate constants are given in $M^{-1}s^{-1}$, produce a free energy of activation that implicitly contains a reference to a standard state for the translational degrees of freedom of 1 M, so is consistent with the free energies as computed in this work.

Table S5. Computed forward (k_f), backward (k_b) rate constants for all the studied reactions. All the other rate constants are given in s^{-1} except for bimolecular reactions, in which the rate constants are given in $M^{-1}s^{-1}$.

Reaction: Bu-9-BBN + $(Me)_2CO$		
Step	k_f	k_b
I1 + CsF ⇋I2 + CsI	6.326E+11	4.609E+11
I2 + Bu9BBN⇋I3	1.993E+10	2.031E+09
I3⇋I4 + F9BBN	1.610E+03	6.433E+04
I4 + $(Me)_2CO$ ⇋I5	6.129E-11	5.697E-14
I5 + CsF⇋I2 + $Cs(OC(Bu)Me_2)$	5.336E+08	6.326E+11

Reaction: Bu-9-BBN + $(CH_2F)_2CO$		
Step	k_f	k_b
I1 + CsF ⇋I2 + CsI	6.326E+11	4.609E+11
I2 + Bu9BBN⇋I3	1.993E+10	2.031E+09
I3⇋I4 + F9BBN	1.610E+03	6.433E+04
I4 + $(CH_2F)_2CO$ ⇋I5	1.993E-05	3.052E-15
I5 + CsF⇋I2 + $Cs(OC(Bu)(CH_2F)_2)$	4.622E+12	6.326E+11

Reaction: Bu-9-BBN + $(CHF_2)_2CO$		
Step	k_f	k_b
I1 + CsF ⇋I2 + CsI	6.326E+11	4.609E+11
I2 + Bu9BBN⇋I3	1.993E+10	2.031E+09
I3⇋I4 + F9BBN	1.610E+03	6.433E+04
I4 + $(CHF_2)_2CO$ ⇋I5	1.574E+02	90659E-15
I5 + CsF⇋I2 + $Cs(OC(Bu)(CHF_2)_2)$	1.113E+14	6.326E+11

Reaction: Bu-9-BBN + $(CF_3)_2CO$		
Step	k_f	k_b
I1 + CsF ⇋I2 + CsI	6.326E+11	4.609E+11
I2 + Bu9BBN⇋I3	1.993E+10	2.031E+09
I3⇋I4 + F9BBN	1.610E+03	6.433E+04
I4 + $(CF_3)_2CO$ ⇋I5	1.061E+06	2.753E-15
I5 + CsF⇋I2 + $Cs(OC(Bu)(CF_3)_2)$	5.822E+14	6.326E+11

References

1. S. Hoops, S. Sahle, R. Gauges, C. Lee, J. Pahle, N. Simus, M. Singhal, L. Xu, P. Mendes and U. Kummer, *Bioinformatics*, **2006**, *22*, 3067-3074.
2. M. von Smoluchowski, *Ann. Phys*., **1906**, *326*, 756-780.
3. (a) CRC Handbook of Chemistry and Physics, CRC Press, Boca Raton, FL, 84th edn., 2003; (b) L.-M. Omota, O. Iulian, O. Ciocîrlan and I. Nită, *Rev. Roum. Chim., **2008**, 53, 977-988.*
4. H. Eyring, *Chem. Rev*., **1935**, *17*, 65-77.
